# Risk Perceptions of Air Pollution and Climate Anxiety in Residents of High Andean Urban Hillsides: A Hierarchical Regression Analysis

**DOI:** 10.3390/ejihpe16090145

**Published:** 2026-09-19

**Authors:** Enma T. Huaman-Chulluncuy, Verónica Luna-Ccoa, Arturo Zuñiga-Blanco, Máximo Córdova Huamaní, Juan Huillca Ochoa

**Affiliations:** 1 School of Psychology, Universidad Continental, Cusco 08001, Peru; 2 Department of Philosophy and Psychology, Universidad San Antonio Abad del Cusco, Cusco 08003, Peru; veronica.luna@unsaac.edu.pe; 3 Department of Mathematics and Statistics, Universidad San Antonio Abad del Cusco, Cusco 08003, Peru; arturo.zuniga@unsaac.edu.pe; 4 College of Education, Universidad San Antonio Abad del Cusco, Cusco 08003, Peru; maximo.cordova@unsaac.edu.pe (M.C.H.); juan.huillca@unsaac.edu.pe (J.H.O.)

**Keywords:** climate anxiety, perceived risk of air pollution, perceived health, urban vulnerability

## Abstract

Risk perception regarding air pollution in Andean urban environments poses significant challenges to psychological well-being, highlighting the emergence of climate anxiety. Despite this, evidence on how cognitive appraisals of environmental and health risks predict this distress in vulnerable communities remains scarce. This study analyzed the explanatory capacity of environmental risk perceptions and perceived health effects on the variance of climate anxiety among residents of hillside communities in an Andean region of Peru. Using a quantitative, cross-sectional, and analytical design, 205 participants selected by quota sampling were assessed. Validated scales were applied to measure risk perception (individual and social), perceived health effects, climate anxiety, and sociodemographic factors. Hierarchical linear regression analyses revealed that individual risk perception, perceived symptoms and discomfort, as well as health concerns and the adoption of preventive measures, exhibited positive and statistically significant associations with climate anxiety, demonstrating greater explanatory power than sociodemographic variables. The final model explained 34.3% of the variance in climate anxiety (*R*^2^ = 0.343; adjusted *R*^2^ = 0.316), representing a moderate explanatory capacity. Furthermore, significant differences were identified based on gender, educational level, and type of residence (native vs. migrant). It is concluded that climate anxiety in vulnerable urban communities is not an abstract concern, but rather an internalized emotional response. This distress is intensified by immediate somatic experience, the proximal interpretation of atmospheric threats, and the subjective experience of risk at the individual level.

## 1. Introduction

Globally, the anthropogenic environmental pollution represents a critical public health crisis, as evidenced by the sustained increase in morbidity and mortality rates directly linked to the effects of climate change (Abdul-Nabi et al., 2025). These multidimensional repercussions follow a progressive trajectory that compromises public health, food security, housing infrastructure, and human safety (Jannah et al., 2025). From a clinical perspective, this phenomenon leads to an exacerbation of the global burden of disease, manifested in an increase in infectious diseases, cardiorespiratory disorders, and cancer (Engelke Da Silva et al., 2025; Kabir et al., 2025; Semenza et al., 2022). Therefore, environmental pollution and climate change are closely related; pollution arises from any chemical, physical, or biological agent that modifies the natural characteristics of the atmosphere (Harsulkar et al., 2023), while climate change refers to long-term shifts in the Earth’s temperatures and weather patterns (Shivanna, 2022).

Similarly, Mental health is particularly vulnerable to the effects of climate change., the progression of which leads to anxiety disorders, acute stress, and depressive symptoms (Burrows et al., 2024; Clayton, 2020). Moreover, evidence shows that living in environments with high levels of environmental pollution not only triggers widespread psychological distress (Tota et al., 2024), but also acts as a critical determinant in shaping population mental health (Gomes et al., 2025; Heinz, 2024). Within this context, the concept of climate anxiety emerges, defined as persistent emotional distress associated with climate change (Hickman et al., 2021). From this perspective, climate anxiety can range from an adaptive response to a manifestation of functional impairment; empirical evidence significantly associates it with poorer psychological health and greater perceived vulnerability to the consequences of climate change (Clayton, 2020).

Likewise, risk perception emerges as a key explanatory mechanism for analyzing the diversity of emotional responses to environmental threats (Wilson et al., 2019) and represents a strong and distinctive predictor of climate anxiety (Reese et al., 2023). His perception is not solely explained by objective data, but is influenced by experiential, affective, cognitive, and sociocultural factors (van der Linden, 2016). Furthermore, accumulated evidence shows that its psychological effects can occur through direct pathways, such as exposure to extreme events; indirect pathways, such as material insecurity, environmental alteration, and community deterioration; and anticipatory or vicarious pathways, associated with a sustained awareness of future threats (Cianconi et al., 2020; Patwary et al., 2024; Walinski et al., 2023).

The majority of the Latin American population is exposed to air with fine particulate matter concentrations exceeding permissible levels, while mountainous regions are among the most sensitive socio-ecological systems to the effects of climate change (Gouveia et al., 2021). In Peru, glacier retreat, irregular precipitation, and more extreme temperatures are complicating the environmental outlook (Brügger et al., 2021). In the city of Cusco, significant levels of air pollutants, such as PM2.5 and PM10, have been recorded in urban hillsides (Poblete et al., 2025). Similarly, it has been documented that air pollution constitutes a public and urban health problem that shapes social and individual risk perceptions (Monge-Rodriguez et al., 2026). Consequently, climate change perceptions stem from high environmental exposure, social and economic vulnerability, and the prevailing inequalities in this region (Azócar et al., 2021; Sapiains et al., 2024).

In the peripheral areas of the city of Cusco, characterized by housing located on slopes and hillsides, the population is exposed to geographical risks, environmental degradation, and high socio-environmental vulnerability (Di Napoli et al., 2023; Kühnl et al., 2023; Ochoa-Sánchez et al., 2025). Evidence indicates that the combination of high population density and air pollution increases the prevalence of mental health problems (Zijlema et al., 2024). Conversely, the presence of greenery and adequate habitability in the residential environment are inversely associated with anxiety symptoms (Xu et al., 2023). However, in these peripheral areas of Cusco, there is a marked scarcity of green spaces, depriving residents of this environmental protective factor.

Furthermore, sociodemographic variables exert a notable influence on environmental risk perception. In particular, gender determines a differential response, with women being more prone to experiencing a more severe emotional impact compared to men (Clayton et al., 2023). Regarding age, evidence indicates that younger age groups experience greater psychological distress (Ayalon & Roy, 2023), while a higher educational level is associated with greater levels of climate concern (Pinho, 2025).

Despite evidence that climate change has severe health repercussions, gaps remain in data and epidemiological studies in developing Latin American countries, reflecting a shortage of public policies addressing this issue (Lawrance et al., 2022), a lack of integrated responses that prioritize mental health (White et al., 2023), and an absence of anxiety as a core focus (Ojala et al., 2021). 

Furthermore, scientific research on climate anxiety has primarily focused on high-income Western countries or sociodemographic conflicts (Coffey et al., 2021; Hickman et al., 2021). Consequently, empirical evidence from developing regions remains limited, especially regarding the integration of air pollution risk perceptions, their perceived health effects, and climate anxiety. Therefore, this study addresses this gap by analyzing the contribution of these specific perceptual dimensions alongside key sociodemographic factors, such as age, gender, and educational level.

Under this premise, climate change has taken a prominent place in contemporary psychological research, as it is recognized that its effects transcend physical and ecosystemic domains to directly influence emotional well-being and social adaptation (Clayton, 2020). Therefore, mental health is no longer a peripheral component of environmental change, but has consolidated as a substantive dimension of its current impacts (Charlson et al., 2021; Clayton & Crandon, 2025). Building on this issue, the present study aims to analyze the explanatory capacity of air pollution risk perceptions on climate anxiety among residents of urban hillside communities in the High Andes of Peru, evaluating the incremental explanatory contribution of its different dimensions through a five-block hierarchical regression model. In this regard, the following general hypothesis is proposed: the dimensions of air pollution risk perception demonstrate a direct and significant explanatory capacity for climate anxiety, providing a significant incremental explanatory percentage (*R*^2^) at each stage of the hierarchical model beyond the controlling effect of sociodemographic variables (Model 1).

## 2. Materials and Methods

### 2.1. Design

The study adopted a quantitative, observational, non-experimental, and cross-sectional design. This methodology is consistent with the proposed hierarchical regression framework for variance explanation, as it allows for the evaluation of the relationship between public risk perceptions and climate anxiety at a specific point in time and in their natural setting, without manipulating the variables. The analytical structure of the study follows a hierarchical explanatory model, assessing the incremental contribution of different blocks of sociodemographic, perceptual, and experiential variables in accounting for the variance in the dependent variable, climate anxiety. This approach enabled the identification of not only direct associations but also the relative weight of each dimension within a progressive multivariate model.

### 2.2. Population and Sample

The study population consisted of residents of urban neighborhoods located on hillsides in an Andean region of Peru, characterized by conditions of socio-environmental vulnerability and a high perceived risk of air pollution. The final sample included 205 participants, selected through non-probability quota sampling based on gender and district criteria. This strategy allowed for capturing a heterogeneous and diverse composition of sociodemographic profiles, considering gender, age, educational level, and resident type (Table 1), suitable for evaluating analytical regression models, acknowledging that it does not constitute a statistically representative sample at the population level.

The selection of the surveyed locations (peri-urban settlements situated on the hillsides and populated hills of Cusco and Santiago) is justified by their geography and structural vulnerability. The inter-Andean basin topography fosters thermal inversion phenomena that trap smog in the valley, making air pollution both visible and perceptible to hillside communities to air pollution (Romero-Lankao et al., 2012). This daily lived experience in a fragile physical and environmental setting accentuates the subjective experience of risk in marginalized peri-urban sectors (Slovic, 1987). Furthermore, non-probability quotas by gender and district were applied to control for recruitment bias in hard-to-reach terrain. Maintaining balanced subgroups (50.2% men vs. 49.8% women and 50.2% Cusco vs. 49.8% Santiago) ensures the sociodemographic variance necessary for the precision of the hierarchical regression model (Hair et al., 2019; Tabachnick & Fidell, 2019).

Inclusion criteria were: (a) being of legal age, (b) residing permanently (at least 6 months) in one of the selected hillside neighborhoods, and (c) voluntarily agreeing to participate in the study by signing an informed consent form. Exclusion criteria included residing in the study area for less than six months and the incomplete or inconsistent completion of the questionnaires. The sample composition reflected a varied distribution in variables such as gender and diversity in educational levels and resident typology (Native/Migrant), factors that were subsequently accounted for in the analysis.

### 2.3. Procedure

Data collection was conducted in the field through the in-person administration of structured self-report questionnaires, with responses recorded on tablet devices. This process took place in community spaces and participants’ residences under controlled conditions, ensuring data confidentiality and the standardization of instructions.

Three primary instruments were utilized for data collection. First, the Air Pollution Risk Perception Scale (Carrasco et al., 2024), which is rated on a 7-point Likert scale ranging from 1 (lowest score) to 7 (highest score). This instrument assesses two dimensions: individual risk perception (e.g., “How often do you worry about the possible negative consequences of air pollution?”) and social risk perception (e.g., “How serious would you rate the current impact of air pollution around the world?”). This scale demonstrated adequate internal consistency in its original validation (*α* = 0.783) and in the current sample *α* = 0.85 and *ω* = 0.86, with *α* = 0.84 for each of its dimensions.

Second, the Perception of the Health Effects of Air Pollution Scale (Deguen et al., 2012) was utilized, which is measured on a 4-point Likert scale (0 = “Never” to 3 = “Always”). This instrument consists of two dimensions: perceived symptoms and discomfort (e.g., “Suffer from nose irritation,” “Have ‘red’ eyes”), and perception of health risks and quality of life, which includes health concerns and the adoption of preventive measures (e.g., “Avoid opening your windows,” “Air your home,” “Think that your quality of life was being degraded”). The scale showed acceptable reliability in its original validation (*α* = 0.779). In the analyzed sample, an overall coefficient of *α* = 0.82 and *ω* = 0.83 was obtained, with *α* = 0.80 for the first dimension and y *α* = 0.82 for the second. These coefficients demonstrated good internal consistency for the overall instrument and its subscales.

Finally, the Climate Anxiety Scale (CASS-S) (Wu et al., 2023) was used to assess emotional responses associated with climate change and their impact on personal well-being. Responses were recorded on a 5-point Likert scale (1 = “Never” to 5 = “Always”). This scale evaluates cognitive-emotional and functional impacts using items such as “My concerns about climate change interfere with my ability to carry out work or academic activities.” It demonstrated adequate internal consistency in its original validation (*α* = 0.772) and optimal reliability in the current sample (*α* = 0.82, *ω* = 0.83). All instruments have prior evidence of validity and have been adapted to the Peruvian context; the obtained reliability coefficients further support the psychometric soundness of the measures used in this study.

Additionally, relevant sociodemographic data were collected, including age (categorized as young adults, adults, and older adults), sex (male/female), educational level (basic education vs. technical or higher education), and residency status (native resident vs. migrant resident). These variables were selected based on their potential influence on risk perception and emotional responses to environmental pollution.

### 2.4. Statistical Analysis

Statistical analyses were conducted using a combined descriptive and inferential approach. In the first phase, variable distributions were examined using measures of central tendency, dispersion, and visual inspection. Because the variables did not meet the assumption of normality, non-parametric tests were employed for between-group comparisons.

To assess differences in risk perception and climate anxiety across categorical variables, Mann–Whitney *U* tests were utilized for two-group comparisons (e.g., sex and educational level), and Kruskal–Wallis tests were applied for comparisons involving more than two groups (e.g., age and length of residence). When significant differences were detected, post hoc comparisons were conducted using Dunn’s test with a Bonferroni correction. Effect sizes were calculated using the rank-biserial correlation coefficient and epsilon-squared, along with 95% confidence intervals (CIs), to quantify the magnitude of the observed differences.

In the second phase, a hierarchical linear regression model was applied to evaluate the explanatory power of the independent variables on climate anxiety. Unlike algorithm-driven automated methods (e.g., stepwise regression), hierarchical regression grants the researcher direct control over determining the block entry sequence a priori, based on an explicit theoretical, temporal, or causal framework (Cohen et al., 2013; Keith, 2019, Pedhazur, 1997). In the current study, the model was structured into five successive blocks progressing from baseline covariates to coping mechanisms (Cohen et al., 2013; Petrocelli, 2003). The first block incorporated sociodemographic variables (age, sex, educational level, and residency status) as baseline controls. This step aimed to isolate their potential confounding effects and account for the variance attributable to individual characteristics prior to evaluating the primary predictors (Cohen et al., 2013; Petrocelli, 2003). The second block introduced individual risk perception, while the third block added perceived social risk; both represent the primary cognitive appraisal of the threat at the personal and community levels (Folkman, 2013). The fourth block included perceived symptoms and discomfort as somatic manifestations of risk (Folkman, 2013). Finally, the fifth block incorporated perceived health risks and preventive measures as higher-order coping responses (Rogers, 1983). This final block evaluates how the perceived severity of health impacts and the adoption of protective behaviors are associated with climate anxiety, isolating their incremental explanatory contribution above and beyond the four previous blocks. This researcher-determined sequence ensured that the unique explanatory increment (*R*^2^) provided by each domain was evaluated independently.

The threshold for statistical significance was set at *p* < 0.05. Changes in the coefficient of determination (*R*^2^) between models were assessed to estimate the incremental contribution of each variable block. Furthermore, unstandardized coefficients (*B*), their standard errors, and *p*-values were analyzed to identify the most salient predictors in the final model.

All statistical analyses were conducted using R (Version 4.5.3) and RStudio (Version 2026.04.0). Data preparation and management were primarily performed using the *dplyr* and *readr* packages. Inferential analyses and effect size estimations were complemented with the *rstatix* and *effectsize* packages. Hierarchical linear regression models were estimated using functions from the base *stats* package, while *sjPlot* and *apaTables* were utilized to organize and format the results. This methodological approach allowed for the evaluation of the explanatory hierarchy of risk perceptions and their relationship with climate anxiety in a context of prolonged perceived environmental exposure.

Prior to interpreting the hierarchical regression models, the primary assumptions of the analysis were evaluated through the examination of residuals and model diagnostics. Linearity, homoscedasticity, residual normality, as well as the absence of multicollinearity and highly influential observations, were all verified. These diagnostics revealed no severe violations that would invalidate the interpretation of the models (see Supplementary Table S1).

### 2.5. Ethical Considerations

This study was approved by the Institutional Research Ethics Committee (CIEI) of the Universidad Cayetano Heredia in Peru (Approval No. CONSTANCIA-CIEI-80-8-25). The research was conducted in strict accordance with the ethical principles for research involving human participants outlined in the Declaration of Helsinki (World Medical Association, 2013), as well as the guidelines on scientific integrity established in the Singapore Statement on Research Integrity (World Conference on Research Integrity, 2010). The principle of autonomy was safeguarded by obtaining written informed consent prior to data collection. The consent form detailed the study’s objectives, the voluntary nature of participation, and the participants’ right to withdraw at any time without penalty. Furthermore, to ensure participant confidentiality and anonymity, responses were coded and personally identifiable information was removed, thereby guaranteeing the secure and restricted handling of the collected data.

## 3. Results

### 3.1. Comparative Analyses

**Figure 1:**
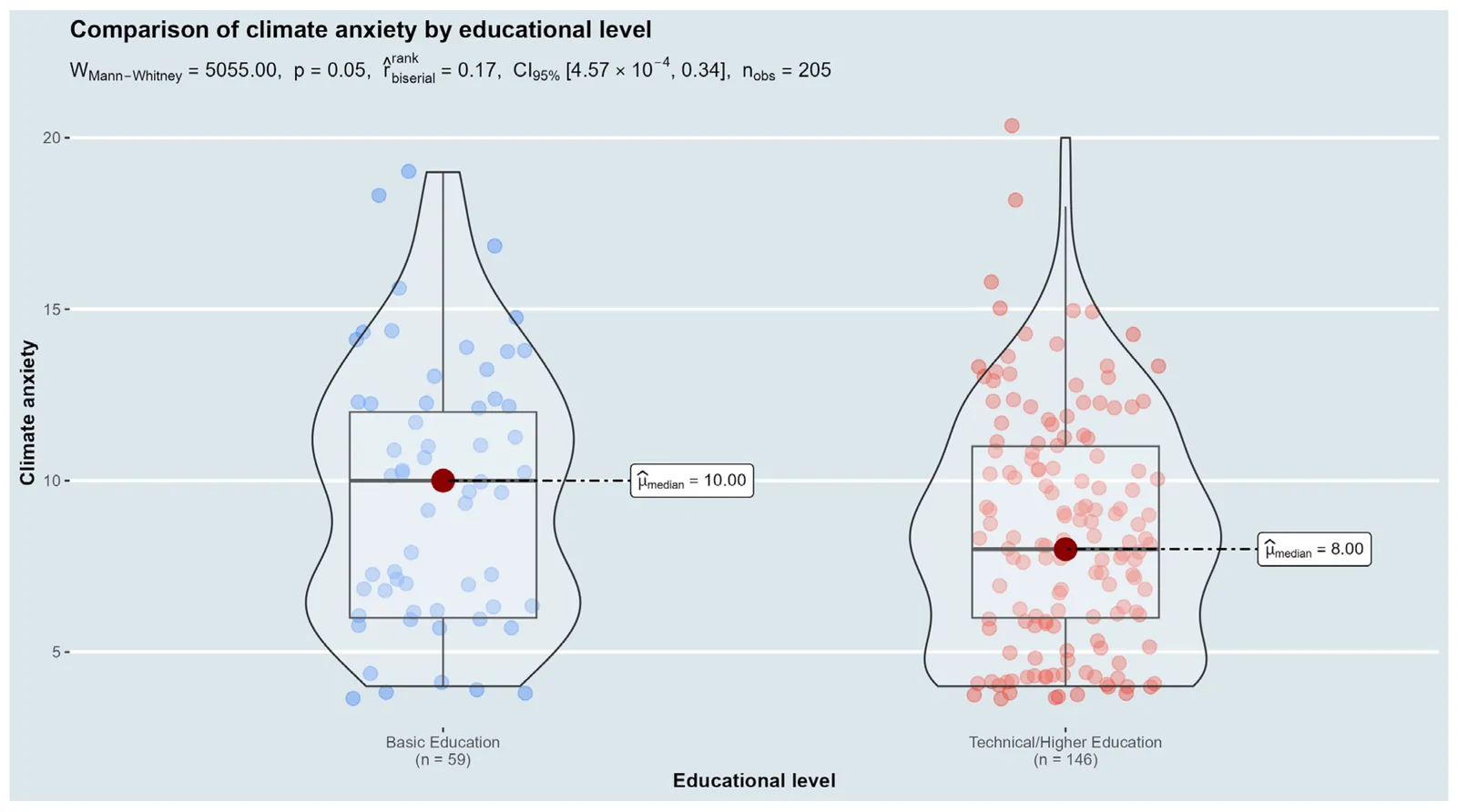
Climate Anxiety by Educational Level.

First, the comparison of climate anxiety by educational level revealed marginally significant differences (Figure 1). The Mann–Whitney *U* test yielded a statistic of *W* = 5055.00, *p* = 0.05, with a small effect size (*r_biserial_* = 0.17, *IC*95% [0.000457, 0.34]). The median climate anxiety score was higher in the group with a basic educational level ($\tilde{x}=10$) compared to those with technical or higher education ($\tilde{x}=8)$. Although dispersion was wide in both groups, the density of values suggested a slight concentration of higher scores in the group with the lowest educational level, indicating greater emotional vulnerability to climate risk within this segment (see Supplementary Table S2).

**Figure 2:**
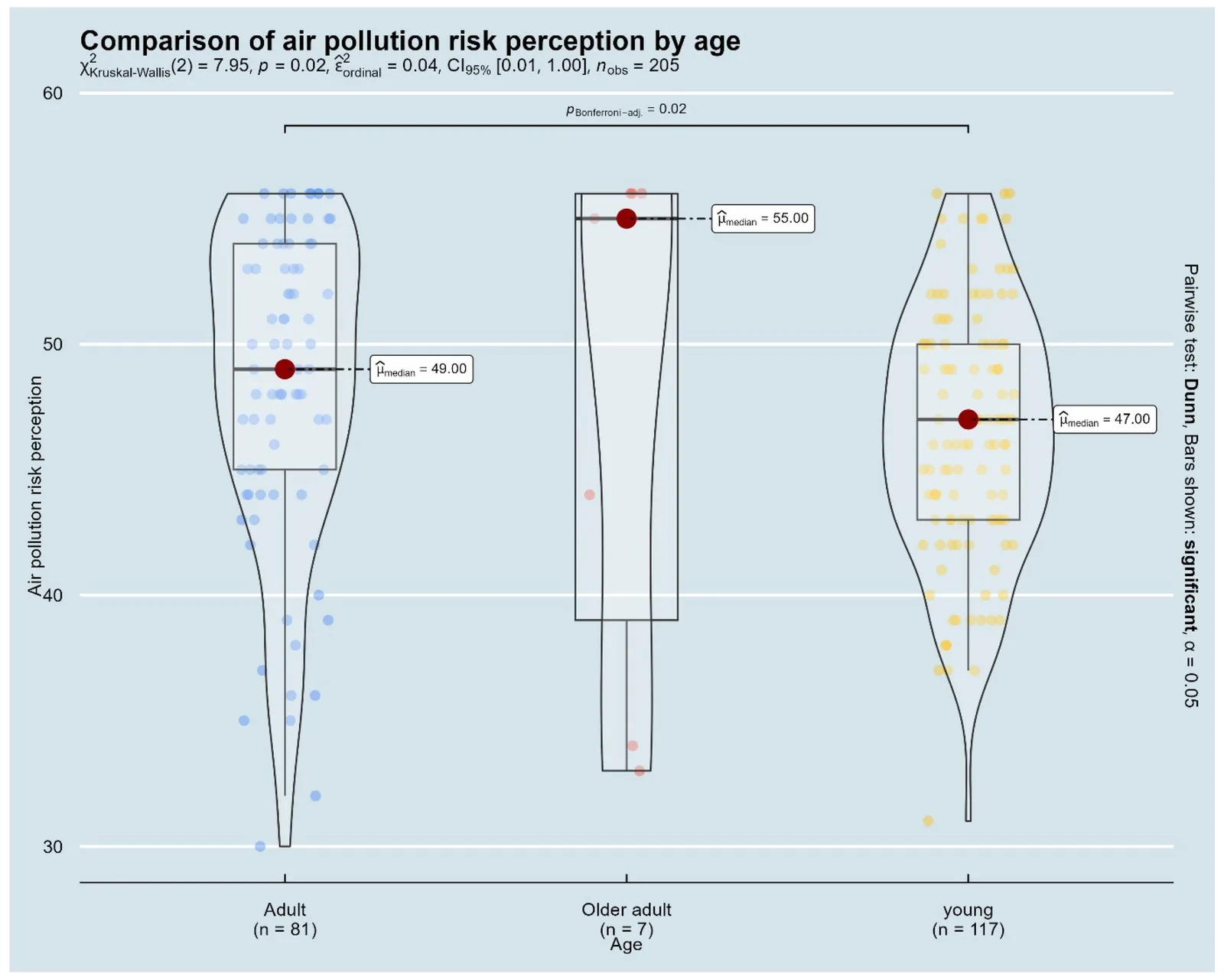
Air Pollution Risk Perception by Age Group.

Regarding air pollution risk perception (APRP), the Kruskal–Wallis analysis by age group revealed statistically significant differences (*χ*^2^(2) = 7.95, *p* = 0.02), albeit with a small effect size (*ε*^2^ = 0.04) (Figure 2). Median scores indicated that older adults exhibited the highest levels ($\tilde{x}=55$), followed by adults ($\tilde{x}=49$) and young adults ($\tilde{x}=47$). However, it should be noted that the older adult group was considerably small (*n* = 7), which limits the robustness of this estimate. Post hoc comparisons (Dunn’s test with a Bonferroni correction) suggested that the overall differences were driven by specific between-group contrasts, although the general trend indicated an increase in risk perception with age (Supplementary Table S2).

**Figure 3:**
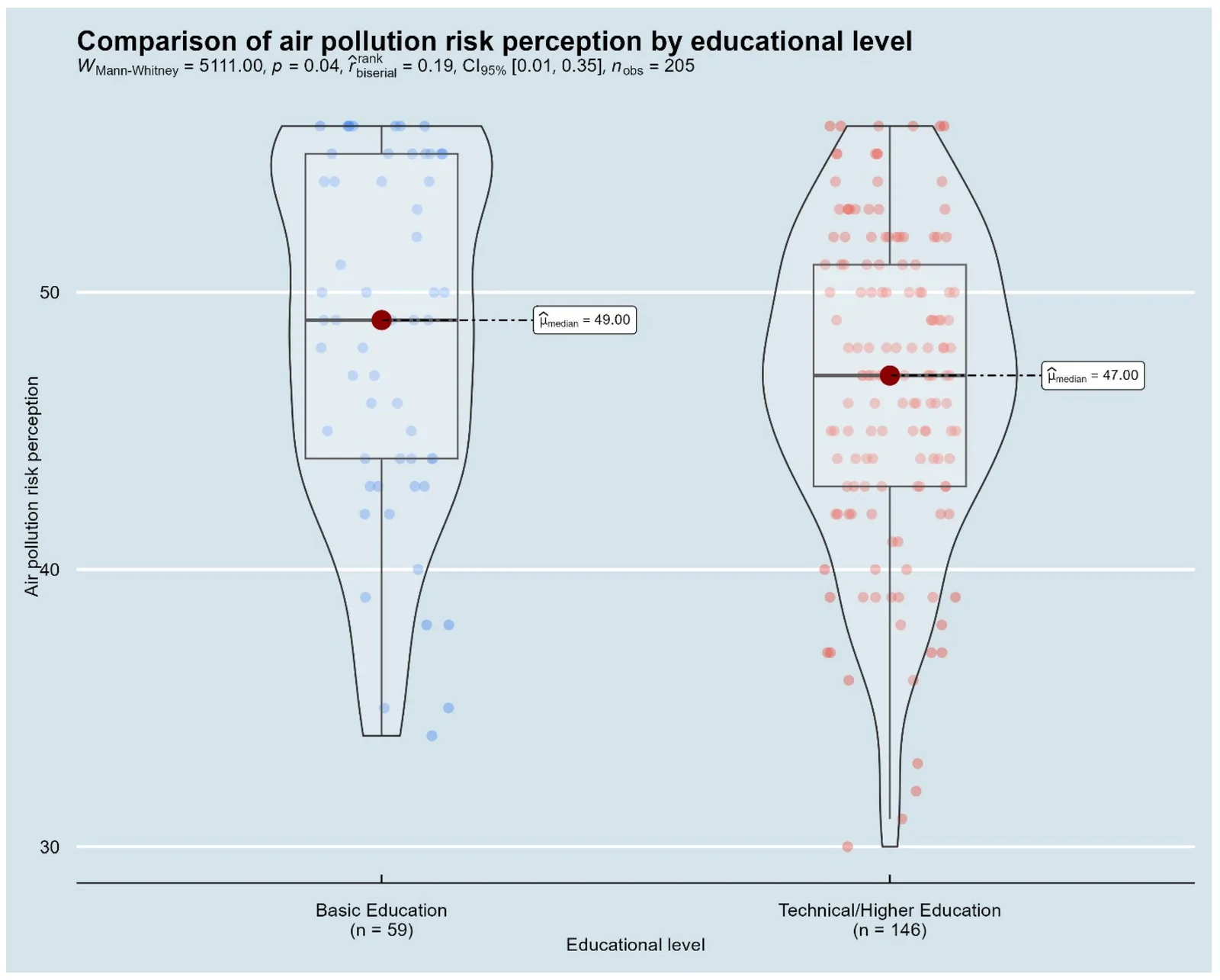
Air Pollution Risk Perception by Educational Level.

Similarly, air pollution risk perception also demonstrated significant differences as a function of educational level (Figure 3). The Mann–Whitney *U* test yielded a statistic of *W* = 5111.00, *p* = 0.04, with a small effect size (*r_biserial_* = 0.19, *IC*95% [0.01, 0.35]). The median score was higher in the basic education group ($\tilde{x}=49$) compared to the group with technical or higher education ($\tilde{x}=47$). This pattern suggests that a lower educational level may be associated with a heightened subjective perception of environmental risk, potentially linked to contextual factors or differential perceived exposure. (see Supplementary Table S2).

**Figure 4:**
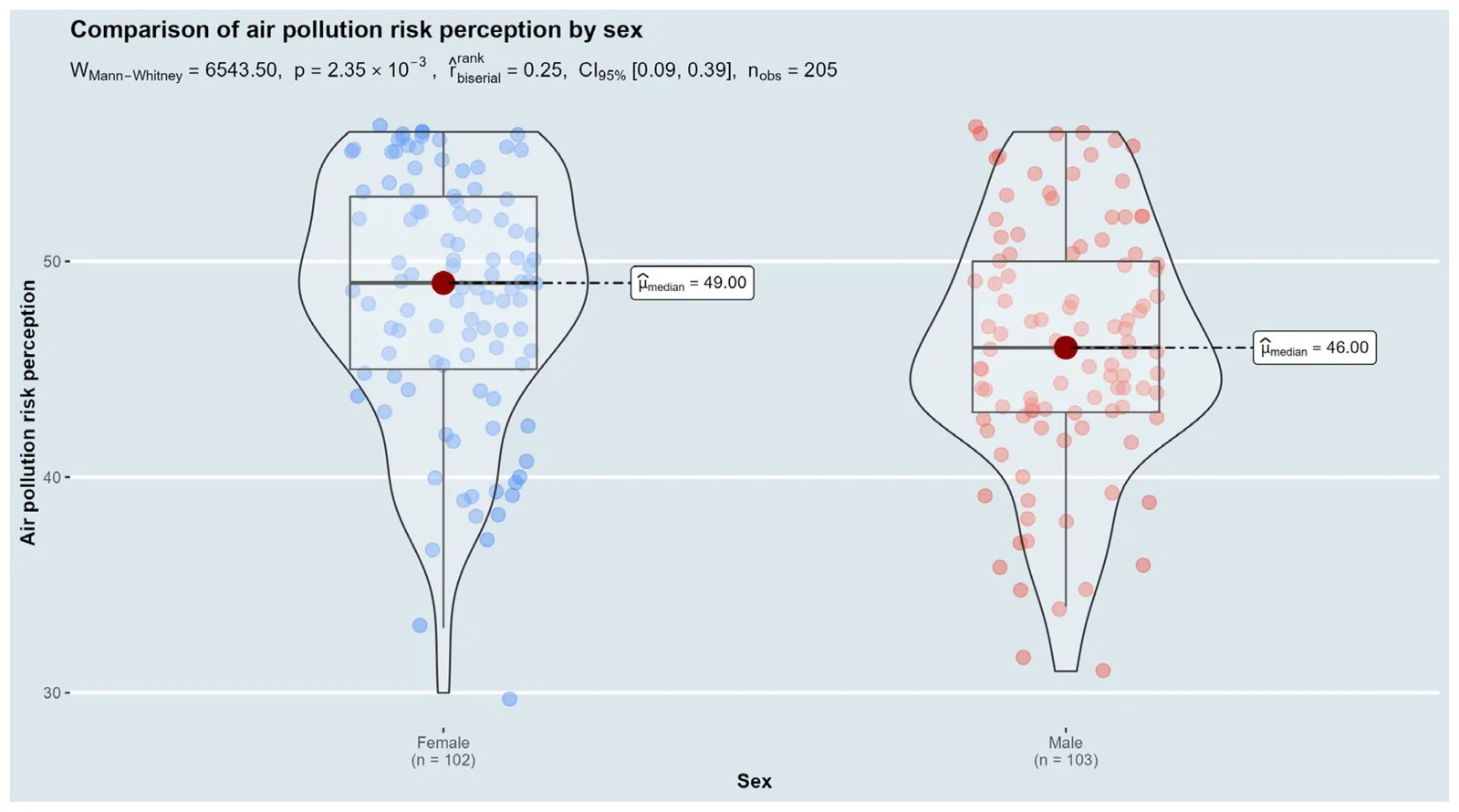
Air Pollution Risk Perception by Sex.

The analysis by sex revealed more pronounced differences in air pollution risk perception (APRP). The Mann–Whitney *U* test indicated that *W* = 6543.50, *p* = 2.35 × 10^−3^, with a small-to-moderate effect size (*r_biserial_* = 0.25, 95 IC % [0.09; 0.39]) (Figure 4). Women exhibited a median APRP score of 49, which was higher than that of men ($\tilde{x}=46$), indicating greater sensitivity to or perception of environmental risk within the female group (Supplementary Table S2).

**Figure 5:**
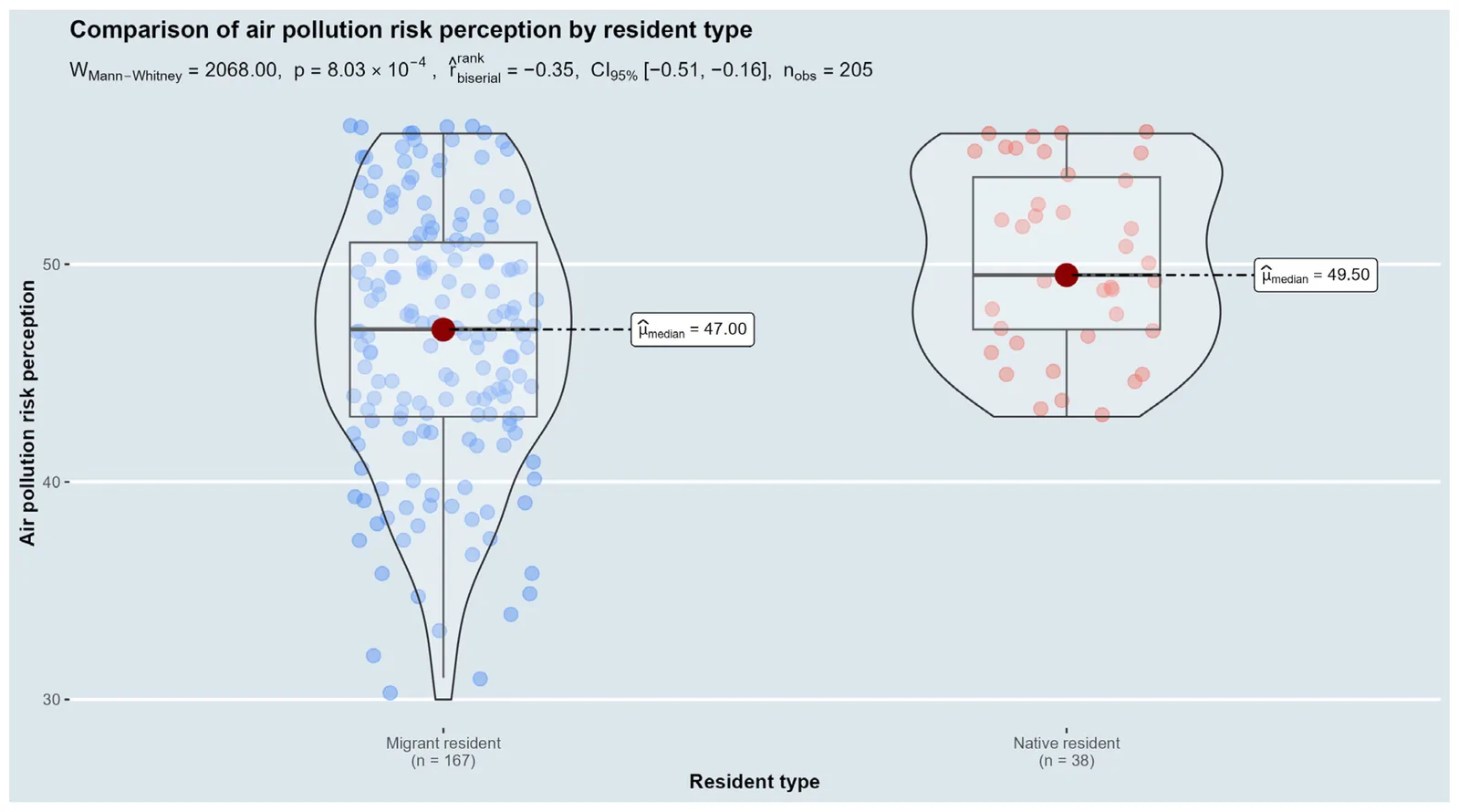
Air Pollution Risk Perception by Residency Status.

Furthermore, Figure 5 demonstrates that air pollution risk perception differed significantly by residency status (Mann–Whitney = 2068.00, *p* = 8.03 × 10^−4^, nobs = 205). Native residents (*n* = 38) reported a higher median risk perception ($\hat{\mu }$_median_ = 49.50) than migrant residents (*n* = 167, $\hat{\mu }$_median_ = 47.00), with a moderate effect size (*r_biserial_* = −0.35, 95 IC % [−0,51; −0,16]). Additionally, the migrant population exhibited considerably greater variability in their responses (range ~ 30–58) compared to a more homogeneous distribution concentrated at higher values among native residents (Supplementary Table S2).

### 3.2. Regression Analysis

A hierarchical regression analysis evaluated the progressive contribution of different variable blocks in explaining climate anxiety (Table 2 and Table 3). Model 1, comprising age, sex, educational level, and residency status, explained 6.1% of the variance (*R*^2^ = 0.061, Adjusted *R*^2^ = 0.042) and was statistically significant, *F*(4, 200) = 3.251, *p* = 0.013. Among the sociodemographic variables, only educational level showed a significant negative association with climate anxiety (β = −0.209, *p* < 0.01), indicating lower levels of climate anxiety as educational level increases.

In Model 2, the inclusion of individual risk perception increased the explained variance to 12.1% (*R*^2^ = 0.121, *R*^2^ adjusted = 0.099). This variable demonstrated a positive association with climate anxiety (β = 0.265, *p* < 0.001), while educational level maintained a negative association (β = −0.173, *p* < 0.05). In Model 3, the addition of social risk perception yielded a minimal increase in explained variance (*R*^2^ = 0.122, *R*^2^ adjusted = 0.095) and did not present a statistically significant association (β = −0.023). Conversely, individual risk perception remained positively associated with climate anxiety (β = 0.277, *p* < 0.01).

The largest increase in explanatory power occurred in Model 4 with the incorporation of perceived symptoms and discomfort, raising the *R*^2^ from 0.122 to 0.301 (*R*^2^ adjusted = 0.276). This variable exhibited the largest standardized association with climate anxiety (β = 0.443, *p* < 0.001). Individual risk perception maintained a smaller positive association (β = 0.165, *p* < 0.05), whereas educational level retained its negative association (β = −0.146, *p* < 0.05).

Model 5 (the final model) explained 34.3% of the variance in climate anxiety (*R*^2^ = 0.343, *R*^2^ adjusted = 0.316), with an additional increment of *R*^2^ adjusted = 0.042. The overall model was statistically significant, *F*(8, 196) = 12.792, *p* < 0.001. Perceived symptoms and discomfort continued to show the largest standardized association (β = 0.333, *p* < 0.001), followed by health concerns and preventive measures (β = 0.244, *p* < 0.001). Educational level maintained a negative association (β = −0.146, *p* < 0.05), while individual and social risk perception, age, sex, and residency status did not show significant associations in the final model.

In summary, explanatory power increased progressively, albeit unevenly across the incorporated blocks. Social risk perception contributed negligible additional variance, whereas perceived symptoms and discomfort produced the largest observed increase. The final model reached a moderate explanatory capacity (*R*^2^ = 0.343); thus, the results imply a moderate explanation of climate anxiety, as approximately 65.7% of its variability remains unaccounted for by the variables considered in the model.

## 4. Discussion

The results reveal a consistent pattern: in this sample of 205 residents from urban hillside communities in an Andean region of Peru, climate anxiety was explained to a limited extent by the initial sociodemographic block, but explanatory power increased significantly upon incorporating perception and environmental health variables. In the final model, individual air pollution risk perception, perceived symptoms and discomfort, as well as health concerns and the adoption of preventive measures, emerged as significant predictors, whereas social risk perception did not contribute additional independent variance. The percentage of explained variance reached a final *R*^2^ of 0.343 and an Adjusted *R*^2^ of 0.316, meaning the model accounts for 34.3% of the variance in climate anxiety. This pattern aligns with traditional hierarchical models that distinguish between distal determinants and proximal antecedents of risk and emotional responses. For instance, van der Linden’s (2016) comprehensive model of climate risk perception, along with subsequent theoretical developments, demonstrates that risk perception, perceived efficacy, and psychological adaptation explain significantly more than demographics alone when attempting to understand complex environmental responses. In this sense, the current study suggests that, within a vulnerable Andean urban context, climate anxiety does not appear to be organized primarily around who the individuals are, but rather around how they interpret and experience perceived environmental risks in their daily lives.

The persistence of individual risk perception in the final model, contrasted with the loss of significance for social risk perception, holds substantial theoretical relevance. This finding is consistent with Psychological Distance Theory (Trope & Liberman, 2010), which posits that phenomena evaluated at a closer distance—in this case, the personal and experiential sphere—are processed through more concrete representations bearing a higher affective load. Consequently, proximal evaluations tend to possess greater explanatory power than collective representations of a distal or abstract nature (Spence et al., 2012). Furthermore, this pattern suggests a reduction in the typical “optimism bias”—a phenomenon wherein individuals tend to perceive environmental threats as a greater problem for society than for themselves (Weinstein, 1980).

In the same vein, van der Linden (2016) argued that personal experience and affect are decisive factors in risk perception; similarly, Leiserowitz (2005) and Weber (2006) emphasized that judgments regarding environmental threats are driven less by a cold calculation of danger than by imagery, emotions, and lived experiences, adding that the perception of environmental changes and the experience of extreme events modify beliefs and adaptive responses. From this perspective, it is understandable that the individual risk dimension, tied to the immediate and daily environment, shows a stronger statistical association with climate anxiety, and plays a more prominent explanatory role than the social dimension. This finding demonstrates that psychological proximity transforms an abstract environmental crisis into a personal threat. Thus, in vulnerable contexts such as urban hillside communities, climate anxiety appears to be fueled by the subjective experience of risk rather than its collective framing.

The explanatory contribution of the dimensions “perceived symptoms and discomfort” and “health concerns and preventive measures” within the hierarchical model further reinforces this interpretation. Literature on environmental risk perception indicates that perceived symptomatology, somatic discomfort, and health concerns are not mere epiphenomena of environmental conditions, but rather central components through which individuals interpret their environment and associate their lived experiences with their emotional states and behaviors. Bickerstaff and Walker (2001) demonstrated that air pollution is experienced and interpreted locally through everyday sociocultural frames; (Day, 2007) emphasized that the experience of air is deeply rooted in place; (Dons et al., 2018; Oltra & Sala, 2018; Reames & Bravo, 2019) showed that risk perceptions and air quality concerns are associated with self-protective behaviors, health concerns, and subjective appraisals of the environment. Moreover, recent reviews have highlighted that subjective measures of pollution capture dimensions that physical-environmental indicators fail to fully account for. Consistent with the results of this study, Gomm and Bernauer (2023) noted that perceived environmental stress outperforms objective indicators in predicting psychological distress, while Bahrami et al. (2024) confirmed that pollution perception is a key pathway for understanding its effects on mental health. Therefore, the data suggest that climate anxiety in this group is directly linked to the perception of physical symptoms and a constant vigilance regarding local health risks.

This interpretation also aligns with current literature on climate anxiety. Building on the work of Clayton (2020) it is now well-established that anxiety responses to the climate crisis combine affective, cognitive, and functional components, and that their intensity depends on both the perceived threat and the resources available to cope with it. Recent empirical evidence indicates that climate anxiety is negatively associated with psychological well-being (Gago et al., 2024). Conversely, it presents positive links with the salience of environmental information, worry, and the adoption of pro-environmental behaviors (Davì et al., 2026; Parmentier et al., 2024); nevertheless, the literature underscores that these relationships are complex, dynamic, and non-linear. Global literature has characterized climate anxiety along two main lines: on one hand, its adverse impact on mental well-being, insomnia, and perceived institutional betrayal (Hickman et al., 2021; Ogunbode et al., 2022, 2023); on the other, its close relationship with information salience, general concern, and pro-environmental commitment (Boluda-Verdú et al., 2022; Whitmarsh et al., 2022). The present study expands on this evidence by demonstrating that, in situations of perceived daily exposure to air pollution, climate anxiety transcends the concern for a global, abstract threat. In this regard, the current findings reveal that climate anxiety is directly fueled by the perception of immediate health harm and somatic-environmental discomfort. This nuance is crucial, as it shifts the debate from a generalized climate anxiety to an “embodied” emotional response anchored in a vulnerable urban environment.

Another noteworthy finding is the role of educational level. In comparative analyses, individuals with basic education tended to exhibit higher medians for both climate anxiety and air pollution risk perception; furthermore, in the hierarchical regression, educational level maintained a negative association with climate anxiety in the final model. This pattern does not necessarily contradict existing literature, but it does introduce an important nuance. Comparative studies on climate risk perception often show that sociodemographic effects exist, though they are weaker and less consistent than cognitive, experiential, and affective factors; van der Linden (2016) explicitly highlighted this, and global findings such as those by Lee et al. (2015) also suggest that education and knowledge can increase awareness and risk perception, but not uniformly across all contexts. In the realm of environmental pollution, Subiza-Pérez et al. (2020) found that education and other contextual variables contribute only modestly compared to more immediate predictors such as sex, age, knowledge, and self-interest concerns. Therefore, the results presented in this study suggest that a higher educational level does not diminish concern about pollution but rather provides analytical and coping tools that prevent this fear from escalating into anxiety. Conversely, a lower educational level may cause the environmental hazard to be perceived as uncontrollable and significantly more threatening in daily life. This aligns perfectly with theories suggesting that climate anxiety stems precisely from uncertainty and a loss of control (Yu et al., 2020).

Differences by sex and residency status are also revealing, though they must be interpreted cautiously, as not all these effects remained independent predictors of climate anxiety in the final regression. In the bivariate comparison, women exhibited higher APRP than men, a finding widely documented in the environmental and climate literature. van der Linden (2016) described a recurring sociodemographic pattern wherein women and young people express greater climate concern; Subiza-Pérez et al. (2020) found sex and age to be among the most stable predictors of perceived environmental risk; and Wullenkord et al. (2021) observed higher levels of climate anxiety among women in a German-speaking sample. In the present study, however, sex did not remain an independent predictor of climate anxiety in the final model, suggesting that part of this difference might be mediated by variations in individual risk perception and health effects. A similar pattern was observed regarding residency status, where the higher risk perception among native residents (individuals born in the area) compared to migrant residents (individuals who migrated to the area at some point in their lives) aligns with studies highlighting the importance of place attachment, cumulative exposure, and localized experiences of environmental and climate degradation. Day (2007), Reames and Bravo (2019), Carlton et al. (2016) and Demski et al. (2017) demonstrate that lived proximity and environmental experience shape risk perception, even if the translation of that perception into psychological outcomes is not always direct. In summary, sex and residency status influence risk perception more clearly than they do climate anxiety itself. This suggests a sequence in which sociodemographic factors initially shape the cognitive interpretation of the threat (risk perception), thereby indirectly affecting the emotional response.

Regarding the strengths and novel aspects of this study, at least three contributions stand out. First, it brings climate anxiety research to a context underrepresented in the literature: residents of Andean urban hillsides experiencing high perceived risk of air pollution and facing cumulative socio-environmental vulnerabilities. Much of the available evidence stems from Global North samples, including young or college-aged populations, and relies on online study designs; in contrast, this study examines climate anxiety in a peripheral urban population in a Peruvian Andean region, where environmental risk perception is deeply intertwined with territorial inequalities. Second, the study bridges two fields that have often advanced separately: climate anxiety literature and research on air pollution risk perceptions and perceived symptoms. Third, it demonstrates that the individual risk dimension and perceived environmental health dimensions explain more variance than social risk perception, thereby helping to refine the distinction between abstract threats and lived threats theoretically. This contribution is particularly relevant in Peru, where previous research has shown that climate perceptions and adaptation priorities are mediated by specific institutional and territorial conditions, and where public communication regarding climate change has historically been conditioned by a context of vulnerability.

Finally, the results support the view that climate anxiety is context-responsive. Recent literature emphasizes that emotional responses to the environmental crisis span a broad spectrum (worry, grief, anger) and should not be classified a priori as dysfunctional. In vulnerable environments, this emotional arousal constitutes a legitimate and adaptive reaction to material reality rather than an individual clinical disorder. Pihkala (2022), Sangervo et al. (2022), Verplanken and Roy (2013) and Verplanken et al. (2020) argue that a certain degree of ecological concern can be adaptive, morally significant, and compatible with environmental engagement, even if it is also associated with distress and functional impairment in specific profiles. Consistent with this literature, the current study suggests that climate anxiety in contexts of chronic air pollution may simultaneously represent an indicator of emotional distress and a form of sensitivity to real environmental threats. The relevance of this finding, therefore, does not lie in medicalizing worry, but rather in demonstrating that when a threat becomes immediate, physical, and health-related, anxiety responses escalate and must be understood as part of the nexus linking environmental degradation, risk perception, and psychological well-being. 

### Limitations

While valuable, the scope of these results is constrained by several methodological factors. First, the study’s cross-sectional design precludes establishing causal relationships or determining temporal directionality between variables. Although the most intuitive pathway suggests that risk perception and perceived health effects trigger climate anxiety, the reverse direction is also plausible: individuals with higher pre-existing anxiety levels may tend to evaluate their environment as more threatening. Second, although the non-probabilistic quota sampling ensured sociodemographic representativeness in hard-to-reach peri-urban areas, it restricts the statistical generalizability of the findings to the broader Andean population. Third, because the data rely on self-reports administered via face-to-face surveys, there is an inherent risk of response biases, such as social desirability toward the interviewer, subjectivity in symptom estimation, or recall bias when reporting the frequency of ailments.

Fourth, the study does not incorporate objective indicators of atmospheric exposure (such as PM_2.5_, PM_10_, or NO_2_ concentrations, spatial dispersion models, traffic density, or proximity to main roads) nor longitudinal measurements. Consequently, the manuscript evaluates the associations between risk perception, perceived symptoms, and climate anxiety, rather than the biological or physical effects of direct exposure. Therefore, it is not possible to accurately estimate the extent to which perceptions reflect actual physical exposure, cumulative exposure, or processes of social interpretation of the environment.

Fifth, by relying exclusively on self-reported data, there is a risk of common method variance. This consideration is crucial in the present study, given that subjective and theoretically closely related constructs—such as risk, symptoms, and anxiety—were analyzed together. Sixth, although the total sample size is adequate for the primary analysis, certain subgroups (specifically older adults in the comparative analyses) are small; thus, some group differences must be interpreted strictly in an exploratory and cautious manner. Finally, the study is confined to a specific geographic context (Andean urban hillsides), which limits its generalizability to other cities, regions, or sociocultural settings.

Nevertheless, these limitations do not invalidate the primary conclusion, wherein the final model demonstrated a moderate and theoretically coherent explanatory power. This justifies its consideration as relevant evidence for future multi-territorial, longitudinal investigations that incorporate georeferenced environmental monitoring.

## 5. Conclusions

Overall, the findings support the conclusion that climate anxiety in highly vulnerable Andean urban contexts is not primarily structured around sociodemographic factors or abstract representations of risk. Rather, it arises from immediate, self-reported appraisals—specifically, individual risk perception and, even more decisively, somatic discomfort and physical symptoms associated with air pollution. This pattern suggests that the psychological experience of risk is firmly anchored in perceived physical symptomatology, health evaluations, and the immediacy of the local environment, thereby shaping a situated emotional response to air pollution rather than a diffuse or purely ideational concern.

The significance of these findings lies in providing empirical evidence from a geographic and social context that remains underrepresented in the scientific literature, thereby contributing to rebalancing the field of climate anxiety research, which has traditionally focused on Western populations and youth samples. By integrating perceptual, experiential, and emotional dimensions into a single analytical model, this study broadens our understanding of climate anxiety as a multidimensional, contextualized phenomenon, demonstrating that its associated factors can vary substantially as a function of the socio-environmental context and subjective risk appraisals.

From an applied perspective, these results suggest that public health interventions and environmental policies should prioritize strategies centered on the local lived experience of risk, addressing perceived symptoms, deploying relatable risk communication, and fostering community-oriented preventive actions. Furthermore, they provide key insights for developing theoretical models that explicitly integrate psychological proximity and embodied experience as central explanatory mechanisms of climate anxiety.

Consequently, these findings offer valuable practical implications and critical avenues for future research. In public management and community health, the results provide empirical evidence for local governments and health agencies to design environmental risk communication strategies that go beyond merely reporting pollution levels, incorporating psychoeducational tools aimed at mitigating emotional distress and climate anxiety in vulnerable hillside communities. Finally, future research should adopt longitudinal designs to clarify the directionality of the observed relationships, incorporate objective measures of environmental exposure (such as the physical monitoring of (*PM*_2.5_) and expand geographic and sociodemographic diversity through probabilistic sampling across other high-Andean regions. Likewise, it is essential to explore contextual mediators and moderators, such as social support networks, community resilience, and access to institutional resources that may buffer or modulate the relationship between risk perception and mental health in environmentally fragile settings.

## Figures and Tables

**Table 1:** Sociodemographic characteristics.

		*f*	%
Sex	Female	102	49.8%
	Male	103	50.2%
Age	Adult	81	39.5%
	Older adult	7	3.4%
	Young	117	57.1%
Level of Education	Basic Education	59	28.8%
	Technical/Higher Education	146	71.2%
Resident type	Migrant resident	167	81.5%
	Native resident	38	18.5%
District	Cusco	103	50.2%
	Santiago	102	49.8%

**Table 2:** Hierarchical Regression Models for Climate Anxiety (Part 1, Models 1–3).

	Model 1		Model 2		Model 3	
	Sociodemographics		Perception of Individual Risk		Perception of Social Risk	
*Predictors*	*B*	*β*	*B*	*β*	*B*	*β*
Age	−0.131	−0.021	−0.39	−0.062	−0.403	−0.064
Sex	0.778	0.109	0.486	0.068	0.494	0.069
Level of education	−0.785 **	−0.209	−0.653 *	−0.173	−0.652 *	−0.173
Resident type	0.665	0.073	0.042	0.005	0.052	0.006
Perception of individual risk			0.239 ***	0.265	0.250 **	0.277
Perception of social risk					−0.03	−0.023
Observations	205		205		205	
R	0.247		0.348		0.349	
*R* ^2^	0.061		0.121		0.122	
Ajusted *R*^2^	0.042		0.099		0.095	
F-statistic	F(4, 200) = 3.251, *p* = 0.013		F(5, 199) = 5.487, *p* < 0.001		F(6, 198) = 4.566, *p* < 0.001	

Nota. B = unstandardized regression coefficient; β = standardized regression coefficient. * *p* < 0.05 ** *p* < 0.01 *** *p* < 0.001.

**Table 3:** Hierarchical Regression Models for Climate Anxiety (Part 2, Models 4 and 5).

	Model 4		Model 5	
	Perceived Symptoms and Discomfort		Health Concerns and Preventive Measures	
*Predictors*	*B*	*β*	*B*	*β*
(Intercept)	0	0	0	0
Age	−0.353	−0.056	−0.115	−0.018
Sex	0.193	0.027	0.138	0.019
Level of education	−0.550 *	−0.146	−0.550 *	−0.146
Resident type	0.643	0.07	0.615	0.067
Perception of individual risk	0.149 *	0.165	0.111	0.123
Perception of social risk	0.006	0.005	−0.02	−0.015
Perceived symptoms and discomfort	0.257 ***	0.443	0.193 ***	0.333
Health concerns and preventive measures			0.202 ***	0.244
Observations	205		205	
R	0.549		0.586	
*R* ^2^	0.301		0.343	
Adjusted *R*^2^	0.276		0.316	
F-statistic	F(7, 197) = 12.134, *p* < 0.001		F(8, 196) = 12.792, *p* < 0.001	

Note. B = unstandardized regression coefficient; β = standardized regression coefficient. * *p* < 0.01 *** *p* < 0.001.

## Data Availability

The data are available upon request from the corresponding author.

## Supplementary Materials

The following supporting information can be downloaded at: https://www.mdpi.com/article/10.3390/ejihpe16090145/s1, Supplementary Table S1. Assumptions check for the final hierarchical regression model. Supplementary Table S2. Descriptive statistics and non-parametric comparisons of climate anxiety and risk perception of air pollution across sociodemographic characteristics.
