# Supplementary material for: Risk Perceptions of Air Pollution and Climate Anxiety in Residents of High Andean Urban Hillsides: A Hierarchical Regression Analysis
Source: Eur J Investig Health Psychol Educ. 2026 Sep 19;16(9):145. doi: 10.3390/ejihpe16090145 (PMC13605110; doi:10.3390/ejihpe16090145)
Supplement: Supplementary file 1 [file ejihpe-16-00145-s001.zip › ejihpe-4454954-supplementary.pdf]

Supplementary Table S1.

Assumptions check for the final hierarchical regression model

Assumption	Procedure / indicator	Result	Interpretation
Normality of residuals	Shapiro–Wilk test	$p = 0.6708$	No evidence of significant deviation from normality of residuals was observed.
Homoscedasticity	Breusch–Pagan test	$p = 0.3876$	No evidence of heteroscedasticity was observed.
Independence of residuals	Durbin–Watson statistic	$DW = 2.064$, $p = 0.6716$	Residuals displayed adequate independence.
Multicollinearity	Variance inflation factor (VIF)	Maximum VIF = 1.66	No multicollinearity issues were observed.
Influential observations	Cook's distance	Maximum Cook's D = 0.064	No highly influential observations were identified.

Note. Assumptions check was performed on the final hierarchical regression model (Model 5).

Supplementary Table S2.

Descriptive statistics and non-parametric comparisons of climate anxiety and risk perception of air pollution across sociodemographic characteristics.

Figure	Educational level	*n*	*Mediana*	IQR	Min.–Max.	Statistic	*p*	Effect size
**Figure 1. Climate anxiety by educational level**	Basic education	59	10.00	6	4-19	W=5055.00	0.05	$r_{biseral}^{kank}$=.17
	Technical/higher education	146	8.00	5	4-20			
	Age group	*n*	*Mediana*	IQR	Min.–Max.	Statistic	*p*	Effect size
**Figure 2. Risk perception o⍰air pollution by age group**	Young adult	117	47.00	7	31-56	$\chi^2_{KW}(2) = 7.95$	0.02	$\hat{\varepsilon}^2_{ordinal} = 0.04$
	Adult	81	49.00	9	30-56			
	Older adult	7	55.00	17	33-56			
	Educational level	*n*	*Mediana*	IQR	Min.–Max.	Statistic	*p*	Effect size
**Figure 3. Risk perception o⍰air pollution by educational level**	Basic education	59	49.00	11	34-56	W=5111	0.04	$r_{biseral}^{kank}$=.19
	Technical/higher education	146	47.00	8	30-56			
	Sex	*n*	*Mediana*	IQR	Min.–Max.	Statistic	*p*	Effect size
**Figure 4. Risk perception o⍰air pollution by sex**	Female	102	49	8	30-56	W=6543.50	0.002	$r_{biseral}^{kank}$=.25
	Male	103	46	7	31-56			
	Resident type	*n*	*Mediana*	IQR	Min.–Max.	Statistic	*p*	Effect size
**Risk perception o⍰air pollution by residency status**	Migrant resident	167	47	8	30-56	W=2068	<0.001	$r_{biseral}^{kank}$=-.35
	Native resident	38	49.5	7	43-56			

Note: W: Mann–Whitney; χ^2_{KW}: chi squared, Kruskall–Wallis.
